# Development of a hybrid alphavirus-SARS-CoV-2 pseudovirion for rapid quantification of neutralization antibodies and antiviral drugs

**DOI:** 10.1016/j.crmeth.2022.100181

**Published:** 2022-02-24

**Authors:** Brian Hetrick, Linda D. Chilin, Sijia He, Deemah Dabbagh, Farhang Alem, Aarthi Narayanan, Alessandra Luchini, Tuanjie Li, Xuefeng Liu, Joshua Copeland, Angela Pak, Tshaka Cunningham, Lance Liotta, Emanuel F. Petricoin, Ali Andalibi, Yuntao Wu

**Affiliations:** 1 Center for Infectious Disease Research, School of Systems Biology, George Mason University, Manassas, VA 20110, USA; 2 Center for Applied Proteomics and Molecular Medicine, George Mason University, Manassas, VA 20110, USA; 3 Department of Pathology, Center for Cell Reprogramming, Georgetown University Medical Center, Washington, DC 20057, USA; 4 Department of Oncology, Lombardi Comprehensive Cancer Center, Georgetown University Medical Center, Washington, DC 20057, USA; 5 TruGenomix, Inc., 155 Gibbs Street, Room 559, Rockville, MD 20850, USA

**Keywords:** Ha-CoV-2, SARS-CoV-2, COVID-19, coronavirus, pseudovirus, lentivirus, mRNA vaccine, SARS-CoV-2 variants, alphavirus, neutralizing antibody, antiviral drug

## Abstract

Severe acute respiratory syndrome coronavirus 2 (SARS-CoV-2) spike protein (S)-pseudotyped viruses are commonly used for quantifying antiviral drugs and neutralizing antibodies. Here, we describe the development of a hybrid alphavirus-SARS-CoV-2 (Ha-CoV-2) pseudovirion, which is a non-replicating SARS-CoV-2 virus-like particle composed of viral structural proteins (S, M, N, and E) and an RNA genome derived from a fast-expressing alphaviral vector. We validated Ha-CoV-2 for rapid quantification of neutralization antibodies, antiviral drugs, and viral variants. In addition, as a proof of concept, we used Ha-CoV-2 to quantify the neutralizing antibodies from an infected and vaccinated individual and found that the one-dose vaccination with Moderna mRNA-1273 greatly increased the anti-serum titer by approximately 6-fold. The post-vaccination serum can neutralize all nine variants tested. These results demonstrate that Ha-CoV-2 can be used as a robust platform for the rapid quantification of neutralizing antibodies against SARS-CoV-2 and its emerging variants.

## Highlights

• Ha-CoV-2 is a non-replicating “hybrid alphavirus-SARS-CoV-2” pseudovirus
• Ha-CoV-2 is composed of SARS-CoV-2 virus-like particle and an alphavirus-based genome
• Ha-CoV-2 can rapidly and robustly express reporter genes in cells within hours
• Ha-CoV-2 is for rapid quantification of viral variants and neutralizing antibodies

## Motivation

Although the SARS-CoV-2 spike-protein-pseudotyped lentivirus or the vesicular stomatitis virus (VSV) has been widely used for the quantification of anti-SARS-CoV-2 neutralizing antibodies and antiviral drugs, these pseudovirions are non-coronavirus-based and require days to express reporter genes. For a robust system, we developed a hybrid alphavirus-SARS-CoV-2 (Ha-CoV-2) pseudovirion. Ha-CoV-2 is a non-replicating SARS-CoV-2 virus-like particle composed of only SARS-CoV-2 structural proteins (S, M, N, and E) and an RNA genome derived from a fast-expressing alphavirus vector. Ha-CoV-2 can rapidly and robustly express reporter genes within 3–6 h, which greatly facilitated its application for the rapid quantification of neutralizing antibodies, viral variants, and antiviral drugs.

## Graphical abstract

**Figure:**
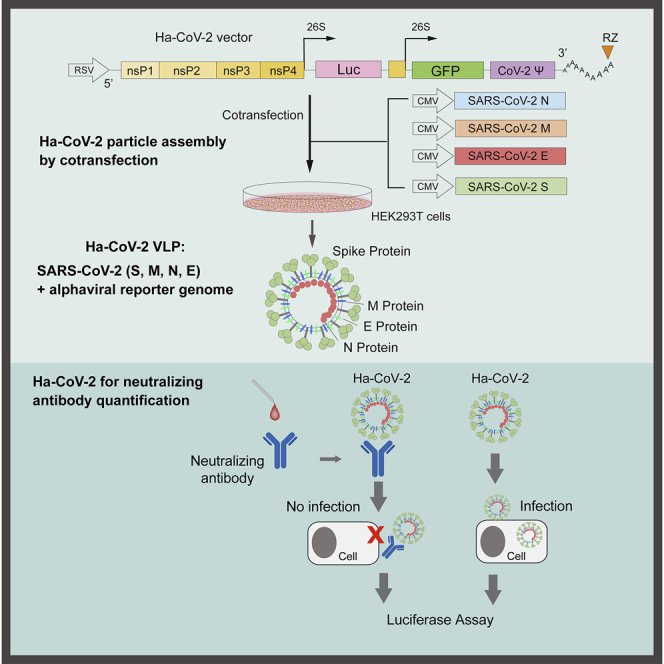

## Teaser

Hetrick et al. develop a hybrid alphavirus-SARS-CoV-2 (Ha-CoV-2) pseudovirion for the rapid and accurate quantification of antiviral drugs, SARS-CoV-2 variants, and their responses to neutralization antibodies. Ha-CoV-2 is a non-replicating SARS-CoV-2 virus-like particle composed of SARS-CoV-2 structural proteins (S, M, N, and E) and a genome derived from a fast-expressing alphavirus.

## Introduction

Severe acute respiratory syndrome coronavirus 2 (SARS-CoV-2) is a rapidly spreading, novel betacoronavirus that is causing the ongoing global pandemic of coronavirus disease 2019 (COVID-19) (Gorbalenya et al., 2020; Wu et al., 2020a, 2020b; Zhou et al., 2020; Zhu et al., 2020). SARS-CoV-2 has caused over 183 million infections and 4 million deaths globally as of July 2021. Antiviral drugs and neutralizing antibodies are effective to combat the pandemic. In particular, neutralizing antibodies, induced by vaccines or by the virus, can play a critical role in controlling and preventing infection. Currently, only one US Food and Drug Administration (FDA)-approved drug, remdesivir, is available to reduce hospital stay (Beigel et al., 2020); several vaccines have recently shown significant results in phase III clinical trials (Jackson et al., 2020; Palacios et al., 2020; Polack et al., 2020; Ramasamy et al., 2021; Voysey et al., 2021) and been approved for emergency use. Nevertheless, the effectiveness of vaccines needs to be continuously monitored for the induction of neutralizing antibodies against evolving viral variants.

Current antiviral drug screening and quantification of neutralizing antibodies rely on the use of SARS-CoV-2 pseudoviruses (Corbett et al., 2020; Dieterle et al., 2020; Nie et al., 2020a, 2020b; Riva et al., 2020; Schmidt et al., 2020; Whitt, 2010; Yang et al., 2020). The use of a live virus requires biosafety level (BSL) 3 facility and practice, which limits large-scale testing and analyses in common laboratories. Both lentivirus and vesicular stomatitis virus (VSV), pseudotyped with the SARS-CoV-2 S protein, are used in cell-based neutralization assays and in antiviral drug screening (Dieterle et al., 2020; Nie et al., 2020a, 2020b; Yang et al., 2020). SARS-CoV-2 contains four structural proteins: the spike (S) protein, the membrane (M) protein, the envelope (E) protein, and the nucleocapsid (N) protein (Siu et al., 2008; Yoshimoto, 2020). S is the major viral protein responsible for virus attachment and entry to target cells (Cai et al., 2020; Huang et al., 2020; Walls et al., 2020) and, thus, is commonly used to pseudotype viruses. Nevertheless, both VSV- and lentiviral-based pseudoviral particles contain only the S protein, and the major structural components are non-SARS-CoV-2 proteins, which may affect virion properties in receptor binding and responses to antibody neutralization (He et al., 2021). In addition, an important issue for the VSV-based pseudovirus is the presence of residual VSV, which can result in high rates of false-positive results (Li et al., 2018). To improve the VSV-based system, highly infectious, recombinant, replication-competent VSV-SARS-CoV-2 viruses have recently been constructed (Case et al., 2020; Dieterle et al., 2020) and shown to express GFP signals as fast as 7.5 h from multiple rounds of viral replication. The systems have been used to quantify neutralizing antibodies and ACE2 inhibitors (Case et al., 2020). Nevertheless, the pathogenic potential of the recombinant, replication-competent VSV-SARS-CoV-2 virus has not been fully investigated, and large-scale production of the new infectious particles may require high bio-containment environments. In addition, the production of viral particles through multiple rounds of viral replication may generate unwanted viral mutations and variants, requiring vigorous screening and validation.

The use of single-entry, non-replicating lenti- or VSV-based pseudoviruses has the safety advantage and a well-controlled course in particle production through DNA transfection. However, for antibody neutralization assays, the use of these particles is time consuming in general, requiring 2 to 3 days to infect and generate reporter signals (Dieterle et al., 2020; Nie et al., 2020a, 2020b; Whitt, 2010; Yang et al., 2020). To overcome the limitations of current pseudoviruses, here we describe the development and validation of a new hybrid alphavirus-SARS-CoV-2 (Ha-CoV-2) particle for the rapid quantification of neutralization antibodies and antiviral drugs. Ha-CoV-2 is a non-replicating SARS-CoV-2 virus-like particle that is composed of authentic virus structural proteins (S, M, N, and E) from SARS-CoV-2, with no structural proteins from other viruses. Ha-CoV-2 also contains a genome derived from an alphavirus-based vector (Ciccarone et al., 1998; Wengler, 1980), which can rapidly and robustly express reporter genes within a few hours of viral entry (Wengler, 1980). In this study, we demonstrate that Ha-CoV-2 can be used as a robust platform for the rapid quantification of neutralization antibodies, viral variants, and antiviral drugs.

## Results

**Figure 1:**
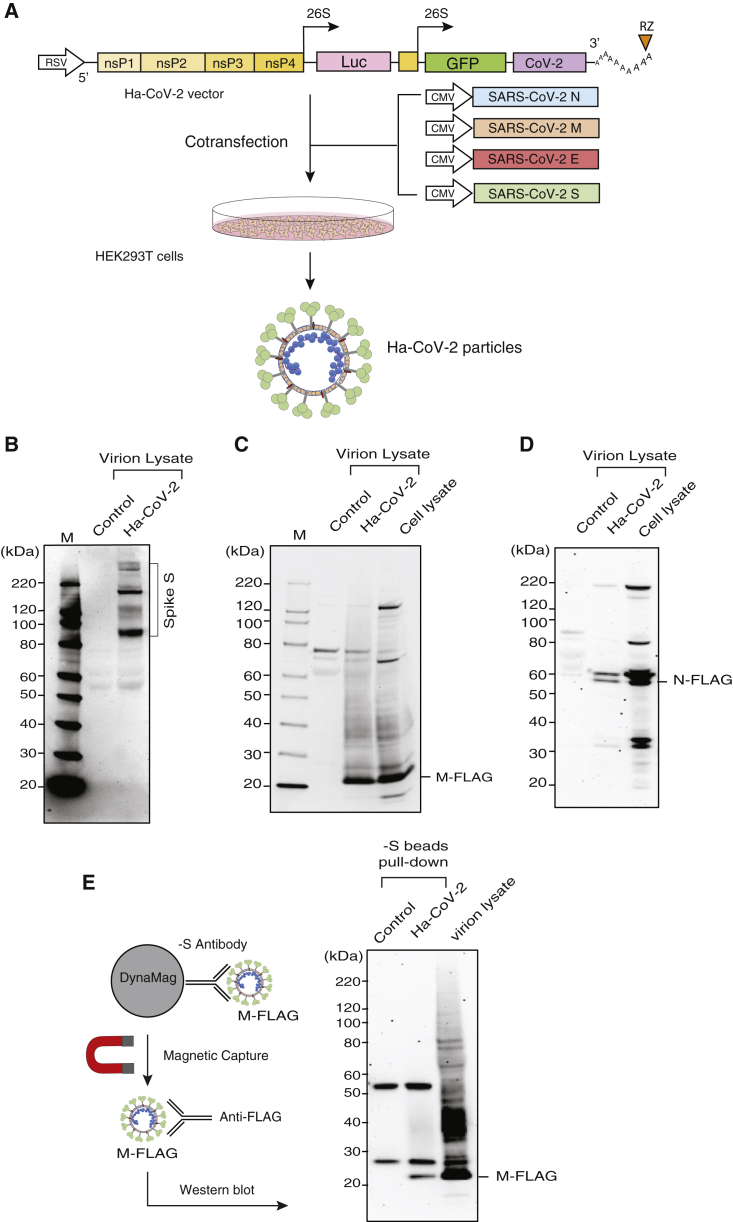
Design and assembly of Ha-CoV-2 particles (A) Illustration of the design of Ha-CoV-2 vector. The vector contains an RSV promoter that transcribes the full-length viral RNA genome to be packaged into Ha-CoV-2 particles. Shown is the 5′ untranslated region followed by open-reading frames coding for nonstructural proteins (nsps) 1–4 from Semliki Forest virus (SFV), viral subgenomic promoters for Luc and GFP reporter expression, the 3′ untranslated region, and a poly(A) tail that is self-cleaved by the hepatitis delta virus ribozyme (RZ). The SARS-CoV-2 packaging signal is inserted in front of the 3′ untranslated region. To assemble viral particles, HEK293T cells were co-transfected with Ha-CoV-2 and the vectors expressing the 4 structural proteins of SARS-CoV-2 (S, M, E, and N). (B) Ha-CoV-2 particles in the supernatant were harvested at 48 h, purified, lysed, and then analyzed by western blot using antibodies for the SARS-CoV-2 S protein. The control is the supernatant from cells transfected with the Ha-CoV-2 vector alone. (C and D) Particles were also assembled using FLAG-tagged M and N. Particles were analyzed with western blot using an antibody against FLAG. (E) Particles in the supernatant were also captured with magnetic beads conjugated with the anti-S antibody and then analyzed with western blot using the antibody again FLAG for FLAG-tagged M protein in the particles.

To establish a rapid cell-based SARS-CoV-2 infection system for the screening and evaluation of neutralizing antibodies and antiviral drugs, we developed a new Ha-CoV-2 viral particle, in which an alphavirus-based RNA genome is enclosed for the rapid expression of reporter genes in target cells (Figure 1A). The genomic RNA consists of the 5′ untranslated region and open-reading frames that code for the nonstructural proteins (nsps) 1–4 from Semliki Forest virus (SFV) (Ciccarone et al., 1998; Liljeström and Garoff, 1991); the inclusion of nsp1–4 allows for self-amplification of the RNA genome in cells. The RNA genome also contains viral subgenomic RNA promoters for the expression of reporter genes (such as firefly luciferase [Luc]). The 3′ end of the genome contains the 3′ untranslated region of SFV and a poly(A) tail that are used to stabilize RNA. In addition, a putative SARS-CoV-2 packaging signal was inserted downstream of the reporter gene to facilitate RNA packaging by the SARS-CoV-2 structural protein N (Hsieh et al., 2005; Lu et al., 2021; Woo et al., 2019). To assemble viral particles, we used the DNA vector Ha-CoV-2 to express the genomic RNA. The Ha-CoV-2 vector was co-transfected with vectors expressing the structural proteins of SARS-CoV-2 (S, M, E, and N) into HEK293T cells (Figure 1A). Virion particles were harvested at 48 h post co-transfection and tested for virion infectivity and the ability to express reporter genes in target cells. First, to confirm the presence of the SARS-CoV-2 structural proteins in Ha-CoV-2 particles, we performed western blots of purified particles using antibodies against the S protein of SARS-CoV-2. We were able to detect the presence of S in Ha-CoV-2 particles (Figure 1B). To further determine the presence of the other structural proteins of SARS-CoV-2, we also assembled Ha-CoV-2 particles using FLAG-tagged M and N proteins and performed western blots using anti-FLAG antibodies. We were able to confirm the presence of both M and N in Ha-CoV-2 particles (Figures 1C and 1D). Furthermore, to determine whether these structural proteins are present in the same virion particles, we used anti-S antibody-conjugated magnetic beads to pull down Ha-CoV-2 particles. The magnetically separated particles were further examined with western blot using the anti-FLAG antibody for the presence of FLAG-tagged M protein. As shown in Figure 1E, we detected the presence of M in the anti-S antibody pull-down particles, confirming that the co-transfection of the SARS-CoV-2 structural proteins with the Ha-CoV-2 vector led to the production of SARS-CoV-2 virus-like particles (VLPs).

**Figure 2:**
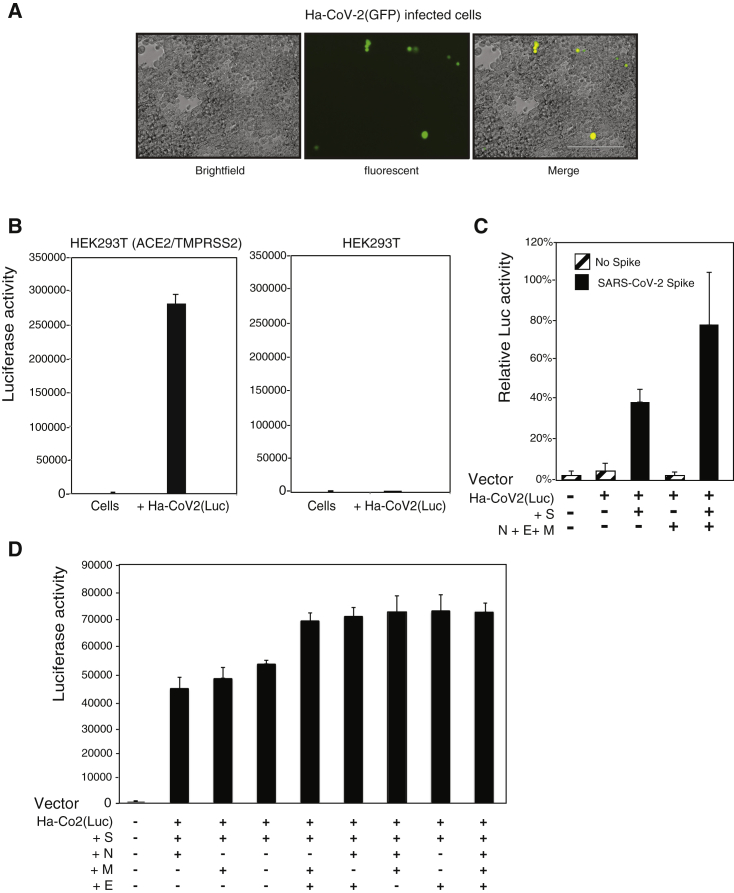
SARS-CoV-2 S protein and ACE2-dependent infection of target cells by Ha-CoV-2 (A) HEK293T(ACE2/TMPRSS2) cells were infected with Ha-CoV-2(GFP) particles. GFP expression was observed 48 h post infection. (B) ACE2-dependent infection of target cells by Ha-CoV-2(Luc). HEK293T(ACE2/TMPRSS2) and HEK293T cells were infected with Ha-CoV-2(Luc) particles. Luciferase expression was quantified at 24 h post infection. (C) SARS-CoV-2 S-protein-dependent infection of target cells by Ha-CoV-2(Luc). Particles were assembled in the presence or absence of S or M + E + N. Luciferase expression was quantified at 4 h post infection. (D) Requirements of M, E, and N for optimal infectivity of Ha-CoV(Luc). Particles were assembled in the presence of S and in combinations of individual proteins of M, E, and N. Luciferase expression was quantified. Infection and luciferase assays in (B)–(D) were performed 3 times, and the mean and standard deviation (SD) are shown.

To further demonstrate the ability of Ha-CoV-2 particles to infect and express reporter genes in target cells, we assembled an Ha-CoV-2(GFP) reporter virus and used it to infect HEK293T(ACE/TEMPRSS2) cells that overexpressed both ACE2 and TMPRSS2. We observed GFP expression in cells following infection (Figure 2A), demonstrating that the alphavirus-based RNA genome can be packaged by the budding SARS-CoV-2 VLPs and that it is capable of expressing the GFP reporter gene in target cells. To determine whether the infection of target cells by Ha-CoV-2 is dependent on the interaction of S with the ACE2 receptor (Li et al., 2003; Zhou et al., 2020), we assembled an Ha-CoV-2(Luc) reporter virus and used it to simultaneously infect HEK293T(ACE/TEMPRSS2) cells and the parent HEK293T cell. As shown in Figure 2B, Ha-CoV-2(Luc) expressed high levels of Luc in the HEK293T(ACE/TEMPRSS2) cells but minimal levels of Luc in HEK293T, demonstrating the requirement of ACE2 for Ha-CoV-2 infection. We further confirmed the requirement for S-ACE2 interaction by generating particles without the S protein. As shown in Figure 2C, in the absence of S, Luc expression was highly diminished, demonstrating the requirement of S for Ha-CoV-2 infection. We also tested the requirement for the other structural proteins, M, N, and E, for Ha-CoV-2 infection. Although these proteins were found to be nonessential, removal of M, N, and E led to a reduction in Ha-CoV-2 infection (Figure 2C). We further investigated the individual contributions of M, N, and E for Ha-CoV-2 infection. It appeared that, in general, particles assembled with two or three of these structural proteins gave rise to a higher level of infection than those with only one protein. However, the presence of S plus E appears to be sufficient for the full infectivity of Ha-CoV-2 (Figure 2D).

**Figure 3:**
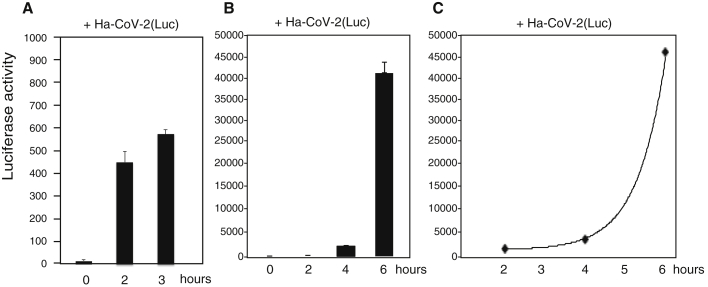
Rapid time course of reporter gene expression in Ha-CoV-2(Luc) infection Time courses of 3 and 6 h of luciferase expression following infection of HEK293T(ACE2/TMPRSS2) cells with Ha-CoV-2(Luc) particles. (A) Cells were infected with Ha-CoV-2(Luc) for 2 and 3 h, washed, and then lysed and analyzed for Luc expression. (B and C) Cells were infected with Ha-CoV-2(Luc) for 2 h, washed, cultured in fresh medium, and then lysed and analyzed for Luc expression at different time points. The addition of virus to cells was defined as time “0”. Infection and luciferase assays were performed 3 times, and the mean and SD are shown.

A major advantage of utilizing alphavirus-based RNA genomes for Ha-CoV-2 is the extremely fast speed and high-level gene expression of alphaviruses; gene expression from the subgenomic RNA promoters occur within hours of infection, and levels of viral plus RNAs can reach 200,000 copies in a single cell (Ciccarone et al., 1998; Wengler, 1980). We followed the time course of Ha-CoV-2(Luc) infection and observed that the Luc reporter expression increased rapidly within 6 h from the addition of particles to cells (Figure 3). This rapid reporter expression kinetics permitted us to utilize Ha-CoV-2 for the fast screening and quantification of neutralization antibodies and antiviral drugs.

**Figure 4:**
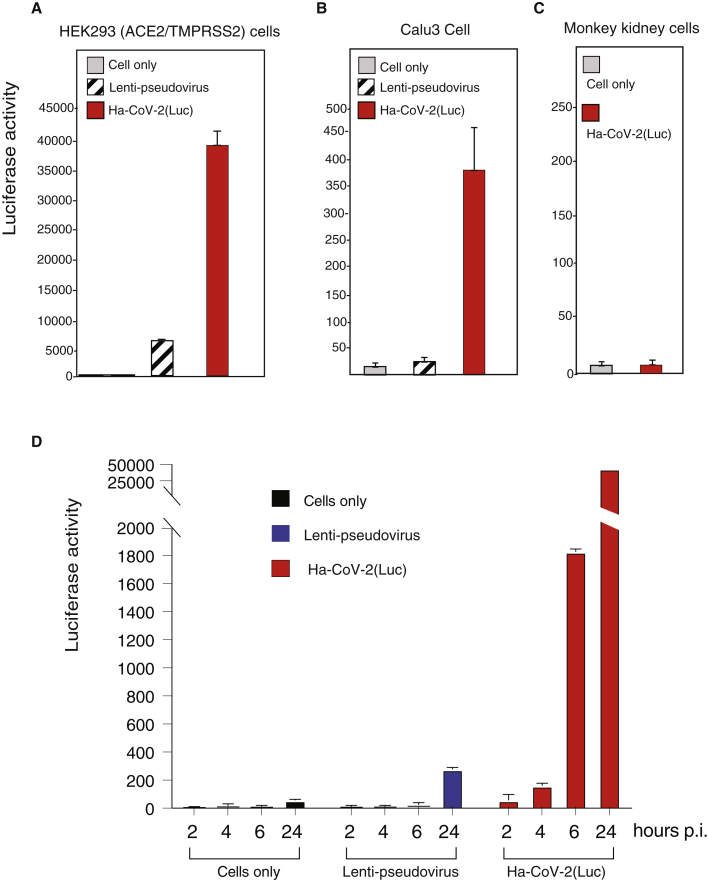
Comparison of the infection time course of Ha-CoV-2 with that of SARS-CoV-2 S-pseudotyped lentivirus (A–C) HEK293T(ACE2/TMPRSS2) and Calu-3 cells were infected with an equal volume of viral particles, Lenti-CoV-2(Luc), or Ha-CoV-2(Luc). Relative infection was quantified by luciferase assay at 72 h post infection. Primary monkey kidney cells were also infected with Ha-CoV-2 for comparison. (D) Comparison of lenti-pseudovirus and Ha-CoV-2 in an infection time course. HEK293T(ACE2/TMPRSS2) cells were infected with an equal volume of viral particles, lenti-CoV-2(Luc), or Ha-CoV-2(Luc). Relative Luc reporter expression was quantified by luciferase assay from 2 to 24 h post infection. Infection and luciferase assays were performed 3 times, and the mean and SD are shown.

Lenti-based SARS-CoV-2 pseudoviruses have been commonly used for antiviral drug screening and neutralization antibody assays (Schmidt et al., 2020; Yang et al., 2020). We performed a comparison of Ha-CoV-2 with lenti-pseudovirus for the infection of both ACE2-overexpressing cells and cells expressing native levels of ACE2. Lenti-pseudovirus and Ha-CoV-2 particles were assembled in similar cell-culture conditions, and an equal volume of the particles was used for infection. Both lenti-pseudovirus and Ha-CoV-2 can infect the ACE2-overexpressing HEK293T(ACE2/TMPRESS2) cells (Figure 4A). However, infection of Calu-3, a human lung cancer cell expressing native levels of ACE2, was minimal with the lenti-pseudovirus (He et al., 2021), whereas Ha-CoV-2 particles produced much higher signals for the infection of Calu-3 cells (Figure 4B). Infection of primary human ACE2-null monkey kidney cells with Ha-CoV-2 did not generate signals above uninfected cell backgrounds (Figure 4C). These results demonstrate that Ha-CoV-2 is likely more sensitive for the infection of low ACE2-expressing cells.

We further followed an infection time course of Ha-CoV-2 and compared it with the infection course of the lenti-pseudovirus. As shown in Figure 4D, in Ha-CoV-2 infection, Luc reporter expression became detectable as early as 2 to 4 h, whereas in the lenti-pseudovirus infection, Luc reporter expression was detectable only after 24 h. In addition, the reporter expression in Ha-CoV-2 infection was much more robust; by 24 h, it reached a level approximately 150-fold higher than that generated from the lenti-pseudovirus infection in our assay system.

**Figure 5:**
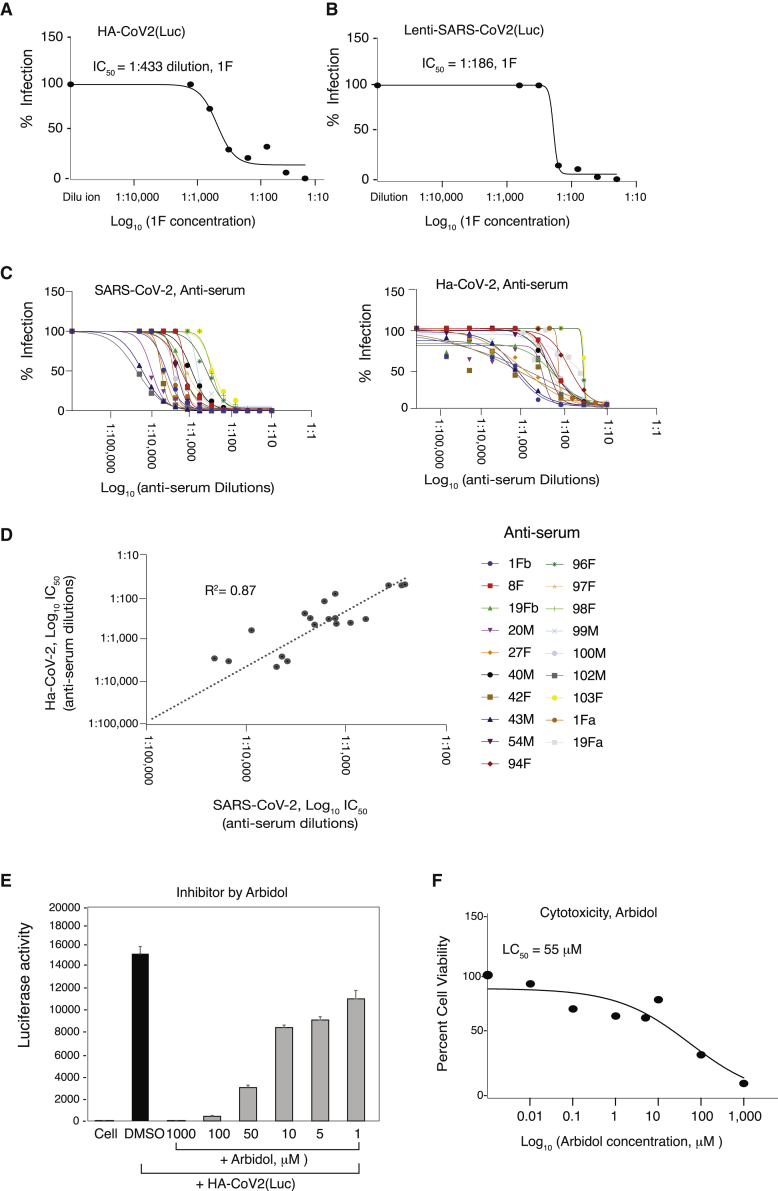
Validation of Ha-CoV-2 particles for rapid screening and quantification of neutralizing antibodies (A) Quantification of neutralizing antibodies with Ha-CoV-2 particles. Shown are the concentration-dependent inhibition of Ha-CoV-2(Luc) by the anti-serum 1F and the 1F inhibition curve. 1F was serially diluted and incubated with Ha-CoV-2(Luc) particles for 1 h at 37°C. The Ha-CoV-2(Luc)-antibody complex was used to infect HEK293T(ACE2/TMPRSS2) cells. Neutralization activities were quantified by luciferase assay at 5 h post addition of virus to cells. Control serum was from healthy, uninfected donors. The IC_50_ was calculated using the relative percentage of infection versus serum concentration. (B) For comparison, the anti-serum 1F was also similarly quantified using a SARS-CoV-2 S-protein-pseudotyped lentivirus, lenti-CoV-2(Luc). Neutralization activities were quantified with luciferase assay at 72 h post infection. (C and D) Correlation of serum neutralization activities quantified with Ha-CoV-2(Luc) and SARS-CoV-2. Convalescent plasma from 19 donors was quantified using infectious SARS-CoV-2 and plaque assays or Ha-CoV-2(Luc). Neutralization activities were plotted, and the IC_50_ values were calculated. The correlation in IC_50_ was plotted. (E and F) Rapid quantification of the anti-SARS-CoV-2 activity of Arbidol. HEK293T(ACE2/TMPRSS2) cells were pretreated for 1 h with Arbidol. Cells were infected with Ha-CoV-2(Luc) in the presence of Arbidol. Viral entry inhibition was quantified by luciferase assay at 5 h. An MTT cytotoxicity assay of Abidol was also performed on cells (F).

To validate Ha-CoV-2 for the rapid screening and quantification of neutralizing antibodies, we tested an anti-SARS-CoV-2 anti-serum (1F), which was serially diluted and pre-incubated with Ha-CoV-2(Luc). The antibody-virus complex was used to infect cells for 5 h for Luc expression. As shown in Figure 5A, we observed 1F concentration-dependent inhibition of Ha-CoV-2(Luc), and the IC_50_ was determined to be at 1:433 dilution (Figure 5A). Given that SARS-CoV-2 lenti-pseudoviruses have been widely used in neutralization assays (Jackson et al., 2020; Nie et al., 2020b; Schmidt et al., 2020), we also performed a similar assay using 1F and a lenti-pseudovirus, lenti-SARS-CoV-2(Luc) (He et al., 2021). Infected cells were analyzed at 72 h post infection. We observed similar 1F concentration-dependent inhibition of the lenti-pseudovirus, and the IC_50_ was found to be at 1:186 dilution (Figure 5B). These results were confirmed using anti-sera from multiple donors (Figure S1) and demonstrated that Ha-CoV-2 is as effective as lenti-pseudoviruses for quantifying neutralizing antibodies but with a much faster speed (5–12 versus 48–72 h).

Based on the 1F results described above, we performed additional validation of Ha-CoV-2-based neutralizing assays using convalescent plasma from 19 donors. The inhibition curve and IC_50_ of each serum are presented in Figure 5C. For comparison, an independent quantification was conducted using infectious SARS-CoV-2 to validate these anti-sera. We observed a direct correlation (r^2^ = 0.87) in the IC_50_ values obtained from Ha-CoV-2 and from SARS-CoV-2 (Figure 5D). These results demonstrated that Ha-CoV-2 can be used for the rapid quantification of neutralizing antibodies.

Pseudoviruses have also been commonly used for high-throughput screening of SARS-CoV-2 entry inhibitors (Whitt, 2010; Yang et al., 2020). We tested a broad-spectrum viral-entry inhibitor, Arbidol (Umifenovir) (Boriskin et al., 2008), for its ability to block Ha-CoV-2(Luc) infection. As shown in Figure 5E, we observed dosage-dependent inhibition of Ha-CoV-2(Luc) in 5 h, and the IC_50_ was determined to be 16 μM. In addition, we also tested a TMPRSS2 protease inhibitor, camostat mesylate, and confirmed that it inhibited Ha-CoV-2 infection of HEK293T(ACE2/TMPRSS2) cells at a high dosage (1 mM) (Figure S2) (Hoffmann et al., 2021). These results demonstrated that Ha-CoV-2 can be used for the rapid screening of SARS-CoV-2 entry inhibitors.

**Figure 6:**
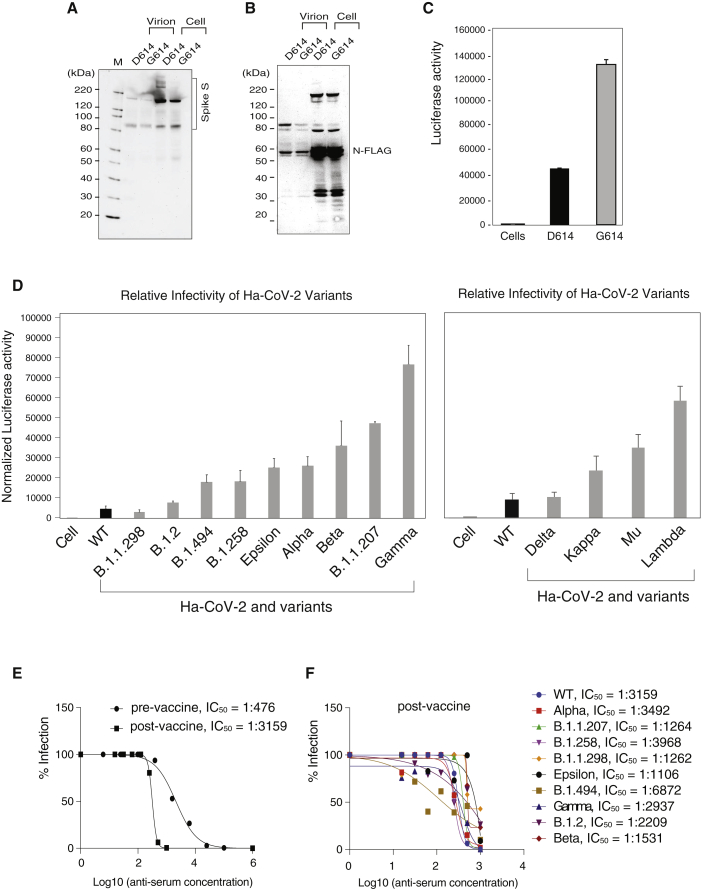
Quantification of the relative infectivity of Ha-CoV-2 variants and their responses to neutralizing antibodies (A and B) Ha-CoV-2(Luc) particles bearing the G614 mutation S or the parent D614 S were assembled and analyzed for the incorporation of S and N in virions. (C) Ha-CoV-2(Luc)(G614) or Ha-CoV-2(D614) was used to infect target cells, and Luc expression was quantified at 5 h. An equal level of viral particles was used for infection. Infection and luciferase assays were performed 3 times, and the mean and SD are shown. (D) A panel of S protein mutants from SARS-CoV-2 variants were used to assemble Ha-CoV-2(Luc) particles and then to infect target cells. The relative infectivity was quantified and normalized with the genomic RNA copies of individual Ha-CoV-2(Luc) variants. Wild type (WT) refers to Ha-CoV-2 derived from the original SARS-CoV-2 strain. Infection and luciferase assays were performed 3 times, and the mean and SD are shown. (E and F) Quantification of anti-serum against Ha-CoV-2(Luc) and its variants. Convalescent plasma from an infected blood donor, before and after one-dose vaccination, was quantified for inhibition of Ha-CoV-2(Luc) infection. Neutralization activities were quantified by luciferase assay at 12 h post infection. The IC_50_ was calculated using the relative percentage of infection versus serum concentration (E). The post-vaccination anti-serum was similarly quantified for the inhibition of Ha-CoV-2(Luc) variants (F).

Finally, we investigated whether the Ha-CoV-2 system can be used for the rapid evaluation of relative infectivity of viral variants. The D614G S mutation emerged early in the COVID-19 pandemic and has recently been reported to confer greater infectivity that has led to the global dominance of the D614G mutant in circulation (Isabel et al., 2020; Korber et al., 2020). To determine whether the increase in virus infectivity can be recapitulated and quantified by the Ha-CoV-2 system, we assembled Ha-CoV-2 particles using the G614 mutant S protein (G614) or the parental S protein (D614). We found that the D614G mutation did not increase virion release or the level of S protein virion incorporation (Figures 6A and 6B). However, Ha-CoV-2 particles bearing the G614 S were found to be nearly 3 times more infectious than those bearing the D614 S (Figure 6C).

We further assembled an additional 13 Ha-CoV-2(Luc) isolates derived from circulating SARS-CoV-2 variants (selected from the GISAID global reference database; Table 1), including Alpha, Beta, Gamma, Delta, Epsilon, Kappa, Lambda, Mu, and several other emerging variants (B.1.1.207, B.1.258, B.1.494, B.1.2, and B.1.1298). Ha-CoV-2(Luc) and the related S protein variants were used to infect target cells, and the relative infectivity was quantified. As shown in Figure 6D, when normalized with the genomic RNA copies, these variants in general are 2- to 10-fold more infectious than the original parental Ha-CoV-2(Luc). These results demonstrated that Ha-CoV-2 can provide a convenient tool for the rapid monitoring and quantification of viral variants. As a proof of concept, we further quantified the ability of an anti-serum to neutralize viral variants. We acquired convalescent plasma from a donor who was infected and then vaccinated with one-dose Moderna mRNA-1273. This one-dose vaccination has greatly increased the anti-serum titer by approximately 6-fold (Figure 6E). Furthermore, when Ha-CoV-2(Luc) variants were tested, we found that the post-vaccination serum had neutralizing activities against all variants tested (Figure 6F). Nevertheless, the neutralizing activities differ greatly among the variants; the anti-serum has the highest neutralizing activity against B.1.494 (IC_50_, 1: 6,872) and the lowest activity against the B.1.1.429 variant (IC_50_, 1:1,106). These results demonstrate that Ha-CoV-2 can be used for the rapid quantification of SARS-CoV-2 variants for potential impacts on neutralizing antibodies and vaccine effectiveness

**Table 1:** List of S protein mutations in SARS-CoV-2 variants

GISAID Variant lineage	Mutations in S protein	GISAID accession number
Alpha	A570D, D614G, D1118H, H69del, N501Y, P681H, S982A, T716I, V70del, Y145del	EPI_ISL_581117
B.1.1.207	D614G, E484K, P681H	EPI_ISL_778908
B.1.1.298	D614G, H69del, I692V, M1229I, V70del, Y453F	EPI_ISL_616802
B.1.2	D614G, E484K, G446V, Y453F	EPI_ISL_833413
B.1.258	D614G, H69del, N439K, V70del	EPI_ISL_755592
Beta	A243del, A701V, D80A, D215G, D614G, E484K, K417N, L242del, L244del, N501Y	EPI_ISL_678597
Epsilon	A222V, D614G, L452R, S13I, W152C	EPI_ISL_847764
B.1.494	A262S, D614G, D796Y, H49Y, L452R, N501Y, P681R, Q613H	EPI_ISL_826591
Gamma	D138Y, D614G, E484K, H655Y, K417T, L18F, N501Y, P26S, R190S, T20N, T1027I, V1176F	EPI_ISL_833136
Delta	Spike T19R, Spike del157/158, Spike L452R, Spike T478K, Spike D614G, Spike P681R, Spike D950N	EPI_ISL_1841283
Kappa	Spike E154K, Spike L452R, Spike E484Q, Spike D614G, Spike P681R, Spike Q1071H	EPI_ISL_1663368
Lambda	Spike G75V, Spike T76I, Spike R246N, Spike del247/253, Spike L452Q, Spike F490S, Spike D614G, Spike T859N	EPI_ISL_2921276
Mu	Spike T95I, Spike Y144S, Spike Y145N, Spike R346K, Spike E484K, Spike N501Y, Spike D614G, Spike P681H, Spike D950N	EPI_ISL_2178402

SARS-CoV-2 lineage identification and variant naming were obtained from GISAID (https://www.gisaid.org). Mutations in the S protein and the sequence accession number of each isolate are listed.

## Discussion

The study of SARS-CoV-2 requires high-level containment that limits the use of infectious viruses in common clinical and research laboratories. Pseudoviruses and VLPs have been widely used for SARS-CoV-2 drug discovery and vaccine development. Pseudoviruses, such as those derived from lentivirus and VSV, can mimic the entry process of SARS-CoV-2. However, structurally, they are very different from SARS-CoV-2 and lack structural components provided by M, E, and N of SARS-CoV-2. VLPs closely resemble SARS-CoV-2 particles, but VLPs contain no genome for reporter expression in target cells (Xu et al., 2020). In this article, we described the development and validation of a novel hybrid system, the Ha-CoV-2 particle, which is structurally a VLP but possesses the ability of a pseudovirus to enter and express reporter genes in target cells.

Ha-CoV-2 also contains a reporter genome derived from the robust expression system of alphavirus, and our results are consistent with previous studies of alphaviral vectors. The high efficiency and rapid gene-expression capacity of alphaviral vectors have been well documented by multiple previous studies. For example, Hahn et al. demonstrated that an alphaviral vector can effectively produce high titer particles (10^8^–10^9^ PFU/mL) in transfected cells and generate >10^6^ chloramphenicol acetyltransferase (CAT) reporter molecules within 7 h after infection of target cells (Hahn et al., 1992). Xiong et al. have also demonstrated that an alphaviral vector can efficiently and rapidly express 10^8^ CAT per cell in 16–20 h (Xiong et al., 1989). Also, gene expression from the subgenomic RNA promoters can occur within hours of infection, and levels of viral plus RNAs can reach 200,000 copies in a single cell (Ciccarone et al., 1998; Wengler, 1980). In addition, alphavirus-based, virus-like vesicles have been used for the rapid and efficient expression of VSV glycolprotein (Kelly et al., 2008; Rolls et al., 1994; Rose et al., 2008, 2014; Schell et al., 2011) and hepatitis B virus antigens (Yarovinsky et al., 2019).

We further demonstrated that Ha-CoV-2 can be used for the rapid screening and quantification of neutralization antibodies, viral variants, and antiviral drugs. We also performed a direct comparison between Ha-CoV-2 and a lenti-pseudovirus in antibody neutralization assays. While both systems are effective in quantifying neutralizing antibodies, the sensitivity of the two systems differs (Figures 5A, 5B, and S2). The lenti-pseudovirus contains only the S protein of SARS-CoV-2, whereas Ha-CoV-2 contains all four structural proteins (S, M, E, and N) of SARS-CoV-2, and has no structural proteins from other viruses (e.g., gag and pol of lentivirus). Although S is the primary requirement for viral entry, the presence of other structural proteins of SARS-CoV-2 may also affect virion infectivity and particle interaction with the cell membrane and antibodies. In our system, the lack of M and E on virion particles does appear to affect virus infection (Figure 2D).

The M glycoprotein in particular is the most abundant protein in SARS-CoV-2 virions, and M has a SemiSWEET sugar-transporter-like structure, suggesting that it may influence the glycosylation of the S protein and S interactions with antibodies (Thomas, 2020). In addition, the N terminus of M in CoVs has a glycosylated ectodomain that protrudes outside the virion and interacts with S, N, and E; it has been shown that SARS-CoV anti-M antibodies can synergize with anti-S and -N antibodies for improved neutralization (Pang et al., 2004; Shi et al., 2006), and M has also been used in protective SARS-CoV and Middle East respiratory syndrome (MERS)-CoV vaccines (Ong et al., 2020). These previous studies suggest that M may affect the neutralizing activities of the anti-S antibody. In our study, we have compared Ha-CoV-2(Luc) (S only) particles with Ha-CoV-2(Luc) (S + M + N + E) particles for their sensitivity to neutralizing antibodies (Figure S3) and found that there was a great correlation (R^2^ = 0.892). However, the Ha-CoV-2(Luc) (S only) particle was found to be slightly more sensitive to antibody neutralization (Figure S3). The mechanism is unknown, but it could be related to the lower infectivity or stability of the Ha-CoV-2(Luc) (S only) particles, which may be more sensitive to inactivation by antibody binding.

We also compared Ha-CoV-2 with infectious SARS-CoV-2 and observed a good direct correlation (R^2^ = 0.87) for antibody quantification, validating that Ha-CoV-2 can serve as a surrogate of SARS-CoV-2 for neutralizing antibody quantification (Figure 5D). However, the SARS-CoV-2-based plaque reduction assay has a higher sensitivity than pseudovirus-based assays in general (Nie et al., 2020a; Yang et al., 2020). One possible reason may be that SARS-CoV-2 virus is produced from infection and productive virus replication and likely contains high percentages of infectious particles, whereas for pseudoviruses, they are assembled by co-transfection in suboptimal conditions with only one or a few SARS-CoV-2 structural proteins and likely contain large amounts of defective particles. Non-infectious defective particles may also bind to antibodies, reducing effective antibody concentrations.

In addition to viral structural proteins, virion particles also incorporate multiple cellular proteins during virion budding and release. Many of these cellular factors, such as PSGL-1, can impact virion infectivity (Fu et al., 2020; He et al., 2021; Liu et al., 2019; Murakami et al., 2020) and antibody binding to the plasma membrane (Umeki et al., 2013). SARS-CoV-2 budding occurs mainly at the membranes of the endoplasmic reticulum (ER)-Golgi intermediate compartment (V’kovski et al., 2020), whereas the lenti-pseudovirus buds from the plasma membrane (Freed, 2015). Because of this difference, it is possible that different sets of cellular proteins may be incorporated into lenti-pseudovirus and SARS-CoV-2. In this regard, the close resemblance of Ha-CoV-2 particles to SARS-CoV-2 may provide a unique tool for studying effects of virion host proteins in SARS-CoV-2 infection and pathogenesis (He et al., 2021).

As SARS-CoV-2 continues to circulate and evolve, viral variants pose a particular challenge for the control of the COVID-19 pandemic, as documented in the recent emergence of the B.1.1.7 lineage in the UK (Rambaut et al., 2020). Viral mutation may lead to increases in viral transmission and fitness, and thus there is an urgent need for the rapid identification and characterization of emerging variants for changes in viral infectivity and responses to neutralizing antibodies. The Ha-CoV-2 system would provide a robust platform for the rapid quantification of viral variants and potential impacts on neutralizing antibodies and vaccine effectiveness.

### Limitations of the study

The present method does have some limitations. For one, Ha-CoV-2-based assays are mostly performed on HEK293T cells that overexpress the human ACE2 and TMPRESS2 receptors. Certainly, human primary cells expressing low levels of ACE2 may require the use of an infection enhancer to acquire robust signals.

## STAR★Methods

### Key resources table

**Table:** 

REAGENT or RESOURCE	SOURCE	IDENTIFIER
**Antibodies**		

SARS/SARS-CoV-2 Spike Protein S2 Monoclonal Antibody (clone 1A9)	Thermo Fisher Scientific	Cat#MA5-35946
DYKDDDDK Tag Monoclonal Antibody ( clone FG4R)	Thermo Fisher Scientific	Cat#MA1-91878
Anti-mouse IgG, HRP-linked Antibody	Cell Signaling	Cat#7076P2

**Bacterial and virus strains**		

SARS-CoV-2 (Isolate USA-WA1/2020)	BEI Bioresources	Cat#NR-52281

**Biological samples**		

Human blood	Healthy adult	N/A

**Chemicals, peptides, and recombinant proteins**		

Arbidol-hydrochloride	Millipore Sigma	Cat#SML0860
Dimethyl sulfoxide	Millipore Sigma	Cat#D8418
Camostat Mesylate	Millipore Sigma	Cat#SML0057

**Critical commercial assays**		

Transfectin Transfection Reagent	Virongy Biosciences	Cat#0.5ml
Dynabeads Pan Mouse IgG	Thermo Fisher Scientific	Cat#11042
Luciferase Assay System	Promega	Cat#E1500

**Deposited data**		

Raw and analyzed data	This paper	N/A

**Experimental models: Cell lines**		

HEK293T cell	ATCC	Cat#CRL-3216
HEK293T(ACE2/TMPRSS2)	Virongy Biosciences	Cat#RCSNAK-01
Calu-3	ATCC	Cat#HTB55

**Recombinant DNA**		

pSARS-CoV-2 S	Sino Biological	N/A
pSARS-CoV-2 S(D614G)	Sino Biological	N/A
pSARS-CoV-2 M	Sino Biological	N/A
pSARS-CoV-2 E	Sino Biological	N/A
pSARS-CoV-2 N	Sino Biological	N/A
pSARS-CoV-2 M-FLAG	Zhang et al. (2020)	N/A
pSARS-CoV-2 N-FLAG	Zhang et al. (2020)	N/A
Ha-CoV-2(GFP)	This study	N/A
Ha-CoV-2(luc)	This study	N/A
pCMVΔR8.2	He et al. (2021)	N/A
pLTR-Tat-IRES-Luc	He et al. (2021)	N/A
pSARS-CoV-2 S (Delta)	Virongy Biosciences	Cat#pCoV2-S**(**B.1.617.2**)**
pSARS-CoV-2 S (Kappa)	Virongy Biosciences	Cat#pCoV2-S**(**B.1.617.1)
pSARS-CoV-2 S (Mu)	Virongy Biosciences	Cat#pCoV2-S(B.1.621)
pSARS-CoV-2 S (Lambda)	Virongy Biosciences	Cat#pCoV2-S(C.37)

**Software and algorithms**		

GraphPad Prism	GraphPad Software	https://www.graphpad.com
FlowJo	Becton Dickinson	https://www.flowjo.com
Adobe Photoshop	Adobe	https://www.adobe.com/products/photoshop.html
Adobe Illustrator	Adobe	https://www.adobe.com/products/illustrator.html

### Resource availability

#### Lead contact

• Further information and requests for resources and reagents should be directed to and will be fulfilled by the Lead Contact, Yuntao Wu (ywu8@gmu.edu).

#### Materials availability

• All unique/stable reagents generated in this study are available from the Lead Contact with a completed Materials Transfer Agreement.

### Experimental model and subject details

The study involved the use of human serum from adult donors. Donor gender identity information was kept confidential per protocols and there is no scientific basis for gender preference in donor selection. Informed consent was obtained from all subjects. All protocols involving human subjects were reviewed and approved by the George Mason University institutional review board (GMU IRB Protocol No. 1592168-9).

### Method details

#### Virus and viral particle assembly

The SARS-CoV-2 S, S(D614G), M, E, or N expression vectors were purchased from Sinobiological. The Ha-CoV-2(Luc) and Ha-CoV-2(GFP) vectors, and the S protein variants were selected from isolates identified in the GISAID global database (Table 1), and synthesized by Twist Bioscience or kindly provided by Virongy Biosciences Inc.. Ha-CoV-2 particles were assembled by cotransfection of HEK293T cells in 10 cm dish with 2.5 μg of each of the SARS-CoV-2 structural protein expression vectors (S, N, E, M) and 10 μg of Ha-CoV-2(Luc) or Ha-CoV-2(GFP). Particles were harvested at 48 hours post cotransfection, filtered through a 0.45 μm filter. Lenti-pseudovirus was assembled by cotransfection of HEK293T cells with SARS-CoV-2 S expression vector (0.5 μg), pCMVΔR8.2 (7.5 μg), and pLTR-Tat-IRES-Luc (10 μg) as previously described (He et al., 2021).

#### Detection of Ha-CoV-2 virion incorporation of structural proteins

The SARS-CoV-2 M-FLAG and N-FLAG vectors were kindly provided by Dr. Pei-Hui Wang (Zhang et al., 2020). HEK293T cells were co-transfected with 10 μg Ha-CoV-2(Luc), 2.5 μg of the SARS-CoV-2 S expression vector, and 2.5 μg each of the M-FLAG, N-FLAG, and E-FLAG vectors. Particles were harvested, filtered through a 0.45 μm filter. Virion lysates were analyzed by SDS-PAGE and western blot using Spike Protein S2 Monoclonal Antibody (1A9) (Invitrogen) (1:1000 dilution) or DYKDDDDK Tag Monoclonal Antibody (FG4R) (Invitrogen) (1: 1000 dilution). Membranes were then incubated with Anti-mouse IgG, HRP-linked Antibody (Cell signaling) (1: 2000 dilution) for 60 min at room temperature. Chemiluminescence signal was detected by using West Pico or West Femto chemiluminescence reagent (Thermo Fisher Scientific). Images were captured with a CCD camera (FluorChem 9900 Imaging Systems) (Alpha Innotech). Particles were also captured with magnetic beads for analyses. Briefly, magnetic Dynabeads Pan Mouse IgG (Invitrogen) (2x10^7^ beads/50 μl) were conjugated with Spike Protein S2 Monoclonal Antibody (1A9) (Invitrogen) (2 μl antibody) for 30 minutes at room temperature. After conjugation, virions were incubated with the anti-S2-beads for 30 minutes at 4°C, and pulled down with a magnet. After washing with cold PBS for 5 times, virions were lysed in LDS lysis buffer (Invitrogen). Lysates were analyzed by SDS-PAGE and western blot using DYKDDDDK Tag Monoclonal Antibody (FG4R) (Invitrogen) (1: 1000 dilution) to detect FLAG-Tagged SARS-CoV-2 M proteins.

#### Viral infectivity assay

Ha-CoV-2 particles were used to infect HEK293T(ACE2/TMPRSS2) cells (a gift from Virongy Biosciences Inc., Manassas, VA), Calu-3 cells (ATCC), HEK293T cells (ATCC) and primary monkey kidney cells provided by Dr. Xuefeng Liu. Briefly, cells were seeded in 12-well plates (2x10^5^ cells per well). Cells were infected for 1-2 hours at 37°C, washed, cultured in fresh medium for 3-48 hours, and then lysed in Luciferase Assay Lysis Buffer (Promega) for luciferase activity using GloMax Discover Microplate Reader (Promega). Lenti-pseudovirus particles were used to infect HEK293T(ACE2/TMPRSS2) cells and Calu-3 cells (ATCC). Cells were infected for 2 hours, cultured for 3 days, and then lysed in Luciferase Assay Lysis Buffer (Promega) for luciferase assays using GloMax Discover Microplate Reader (Promega).

#### Neutralizing antibody assay

Ha-CoV-2 particles were pre-incubated with serially diluted sera from COVID19 patients for 1 hour, and then added to HEK293T(ACE2/TMPRSS2) cells for 2 hours. Cells were then washed, and cultured in fresh medium for additional 3-24 hours. Cells were lysed in Luciferase Assay Lysis Buffer (Promega) for luciferase assays using GloMax Discover Microplate Reader (Promega). For neutralization assays using wild-type SARS-CoV-2 virus, anti-serum was serially diluted (a twelve-point, two-fold dilution series starting at 1:10 dilution), and pre-incubated with 100 pfu of SARS-CoV-2 for 1 hour at 37°C. After incubation, viral plaque assay was conducted to quantify viral titers. Briefly, Vero cells (ATCC) in 12-well plates (2x10^5^ cells per well) were infected with virus for 1 hour at 37°C. After infection, a 1:1 overlay, consisting of 0.6% agarose and 2X EMEM without phenol red (Quality Biological), supplemented with 10% fetal bovine serum (FBS) (Gibco), was added to each well. Plates were incubated at 37°C for 48 hours. Cells were fixed with 10% formaldehyde for 1 hour at room temperature, and then the agarose overlay was removed. Cells were stained with crystal violet (1% CV w/v in a 20% ethanol solution). Viral titer of SARS-CoV-2 was determined by counting the number of plaques.

#### Antiviral drug assay

Arbidol-hydrochloride (Sigma) was resuspended in Dimethyl sulfoxide (Sigma). HEK293T(ACE2/TMPRSS2) cells were pretreated for 1 hour with serially diluted Arbidol. Ha-CoV-2 particles were added to cells, followed by the addition of Abidol to maintain the drug concentration. Cells were infected in the presence of Arbidol for 2 hours, washed, and then cultured in fresh medium for a total of 5 hours. Cells were lysed in Luciferase Assay Lysis Buffer (Promega) for luciferase assays using GloMax Discover Microplate Reader (Promega).

Camostat Mesylate (Millipore Sigma) was resuspended in Dimethyl sulfoxide (Millipore Sigma). Cells were pretreated for 1 hour with serially diluted Camostat Mesylate. Ha-CoV-2 particles were added to cells, followed by the addition of Camostat Mesylate to maintain the drug concentration. Cells were infected in the presence of Camostat Mesylate for 18 hours. Cells were lysed in Luciferase Assay Lysis Buffer (GoldBio) for luciferase assays using GloMax Discover Microplate Reader.

### Quantification and statistical analysis

The study contains a large number of direct quantification of assay values (luciferase activity quantified by luciferase assay reading), but only basic statistic concept (mean and standard deviation) were used to describe these results. Infection and luciferase assays were performed in triplicate as indicated in the figure legends (Figures 2, 3, 4, and 6). Luciferase expression was quantified with luciferase assay and the mean was the average value of the three luciferase assay readings. Standard deviation (SD) were determined using Microsoft Excel. Antibody neutralization activity was plotted using GraphPad Prism 7 and the IC_50_ values were calculated using GraphPad Prism 7.

## Data Availability

• All data generated or analyzed during this study are included in this article. Data reported in this paper will be shared by the lead contact upon request. • This paper does not report original code. • Any additional information required to reanalyze the data reported in this paper is available from the lead contact upon request.

## Supplemental information

Document S1. Figures S1–S3

Document S2. Article plus supplemental information
