# Supplementary material for: Development of a hybrid alphavirus-SARS-CoV-2 pseudovirion for rapid quantification of neutralization antibodies and antiviral drugs
Source: Cell Rep Methods. 2022 Feb 24;2(3):100181. doi: 10.1016/j.crmeth.2022.100181 (PMC8866097; doi:10.1016/j.crmeth.2022.100181)
Supplement: Document S1. Figures S1–S3 [file mmc1.pdf]

Cell Reports Methods, Volume *2*

Supplemental information

Development of a hybrid alphavirus-SARS-CoV-2 pseudovirion for rapid quantification of neutralization antibodies and antiviral drugs

Brian Hetrick, Linda D. Chilin, Sijia He, Deemah Dabbagh, Farhang Alem, Aarthi Narayanan, Alessandra Luchini, Tuanjie Li, Xuefeng Liu, Joshua Copeland, Angela Pak, Tshaka Cunningham, Lance Liotta, Emanuel F. Petricoin, Ali Andalibi, and Yuntao Wu

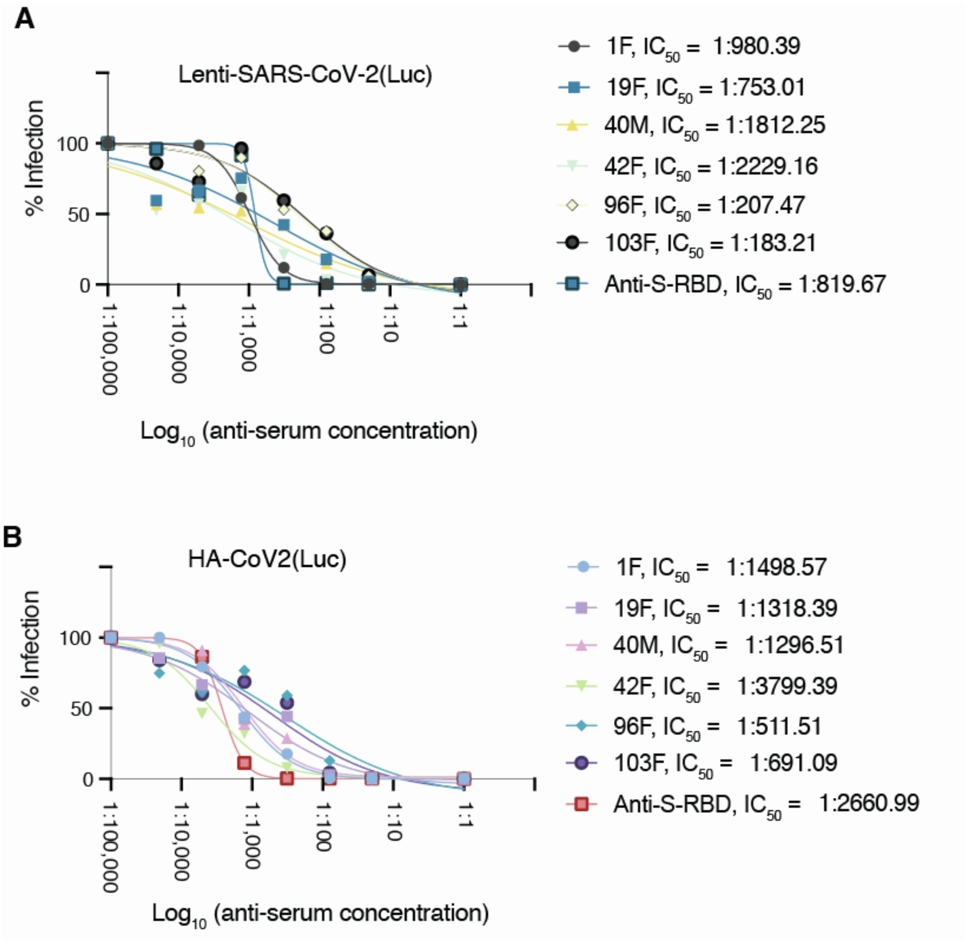

Figure S1. Quantification of neutralizing antibodies with Ha-CoV-2 and a SARS-CoV-2 S protein pseudotyped lentivirus, Lenti-SARS-CoV-2(Luc), Related to Figure 5. (A) Quantification of neutralizing antibodies with Lenti-SARS-CoV-2(Luc) particles. Shown are the concentration-dependent inhibition by anti-sera, which were serially diluted and incubated with Lenti-SARS-CoV-2(Luc) particles for 1 hour at 37°C. The Lenti-SARS-CoV-2(Luc)-antibody complex was used to infect HEK293T(ACE2/TMPRSS2) cells for 18 hours. Cells were washed, and cultured for 48 hours. Neutralization activities were quantified by luciferase assay at 48 hours post addition of virus to cells. Control serum was a monoclonal rabbit anti-SARS-CoV-2 S protein RBD antibody (kindly provided by Virongy Biosciences Inc). **(B)** Quantification of neutralizing antibodies with Ha-CoV-2 particles. Shown are the concentration-dependent inhibition by anti-sera, which were serially diluted and incubated with Ha-CoV-2(Luc) particles for 1 hour at 37°C. The Ha-CoV-2(Luc)-antibody complex was used to infect HEK293T(ACE2/TMPRSS2) cells. Neutralization activities were quantified by luciferase assay at 18 hours post addition of virus to cells. Control serum was the same monoclonal rabbit anti-SARS-CoV-2 S protein RBD antibody described above.

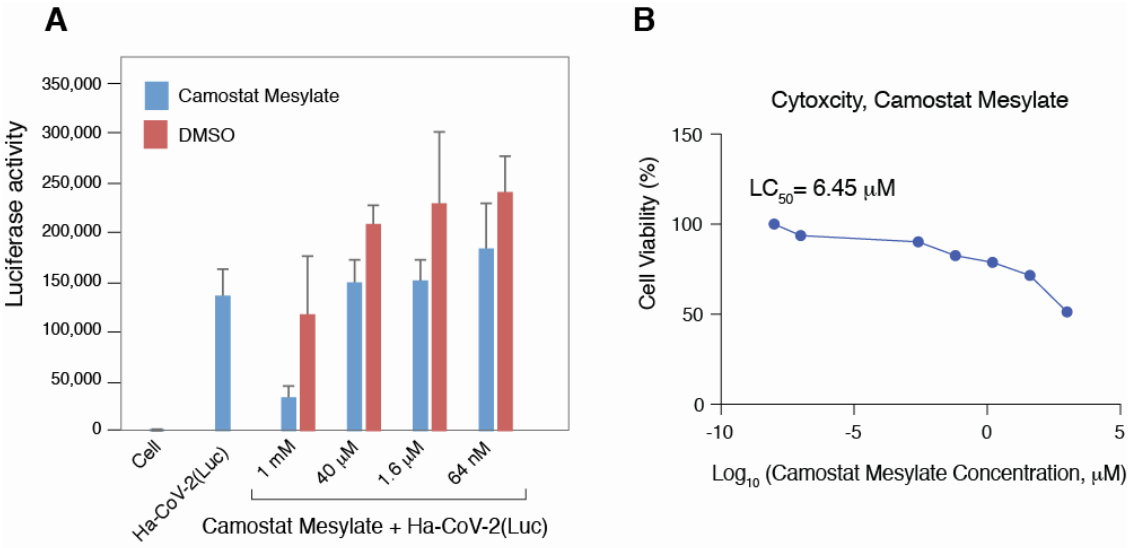

Figure S2. Rapid quantification of the anti-SARS-CoV-2 activity of camostat mesylate, Related to Figure 5. (**A**) HEK293T(ACE2/TMPRSS2) cells were pretreated for 1 hour with camostat mesylate or DMSO at different diluted dosages. Cells were infected with Ha-CoV-2(Luc) in the presence of camostat mesylate. Viral entry inhibition was quantified by luciferase assay at 18 hours. **(B)** An MTT cytotoxicity assay of camostat mesylate on cells was also performed.

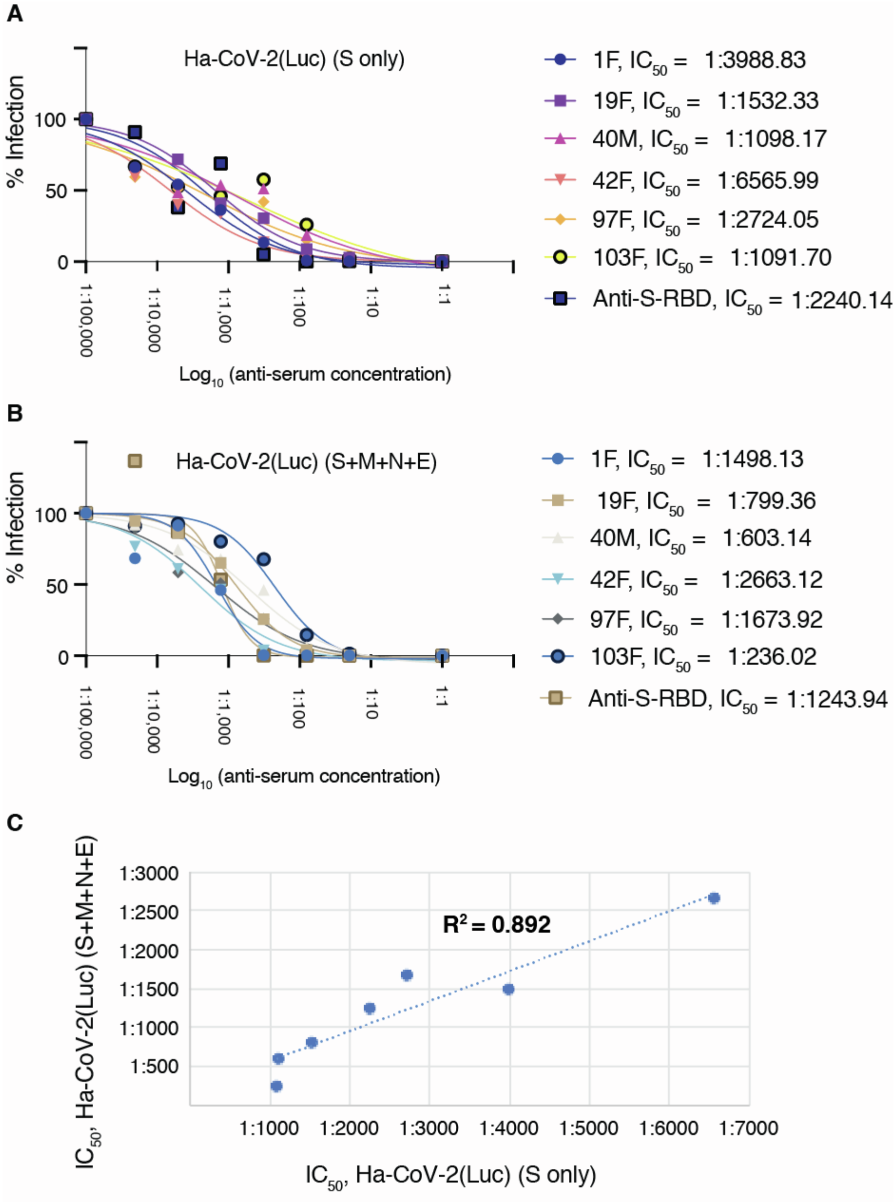

Figure S3. Quantification of neutralizing antibodies with Ha-CoV-2(Luc) (S only) and with Ha-CoV-2(Luc) (S+M+N+E) particles, Related to Figure 2. Ha-CoV-2(Luc) particles were assembled with SARS-CoV-2 S protein or with S + M + N + E proteins, and then used for neutralizing antibody assays. (**A**) Quantification of neutralizing antibodies with Ha-CoV-2(Luc) (S only) particles. Shown are the concentration-dependent inhibition curves by the anti-sera, which were serially diluted and incubated with Ha-CoV-2(Luc) particles for 1 hour at 37°C. The Ha-CoV-2(Luc)-antibody complex was used to infect HEK293T(ACE2/TMPRSS2) cells. Neutralization activities were quantified by luciferase assay at 18 hours post addition of virus to cells. Control serum was a monoclonal rabbit anti-SARS-CoV-2 S protein RBD antibody. (**B**) Quantification of neutralizing antibodies with Ha-CoV-2(Luc) (S+M+N+E) particles. Shown are the concentration-dependent inhibition curves by the anti-sera, which were serially diluted and incubated with Ha-CoV-2(Luc) (S+M+N+E) particles for 1 hour at 37°C. The Ha-CoV-2(Luc)-antibody complex was used to infect HEK293T(ACE2/TMPRSS2) cells. Neutralization activities were quantified by luciferase assay at 18 hours post addition of virus to cells. Control serum was a monoclonal rabbit anti-SARS-CoV-2 S protein RBD antibody. (**C**) Correlation of serum neutralization activities quantified with Ha-CoV-2(Luc) (S only) and SARS-CoV-2(Luc) (S+M+N+E). The IC_{50} values from (**A**) and (**B**) were calculated and plotted for correlation ($R^2 = 0.892$).
